# An Overview of Image Generation of Industrial Surface Defects

**DOI:** 10.3390/s23198160

**Published:** 2023-09-28

**Authors:** Xiaopin Zhong, Junwei Zhu, Weixiang Liu, Chongxin Hu, Yuanlong Deng, Zongze Wu

**Affiliations:** 1 College of Mechatronics and Control Engineering, Shenzhen University, Nanhai Ave., Shenzhen 518060, China; xzhong@szu.edu.cn (X.Z.); zhujunwei21@email.szu.edu.cn (J.Z.); huchongxin2022@email.szu.edu.cn (C.H.); zzwu@szu.edu.cn (Z.W.); 2 Shenzhen Institute of Technology, Jiangjunmao Road, Shenzhen 518116, China; 3 Guangdong Artificial Intelligence and Digital Economy Laboratory (Shenzhen), Kelian Road, Shenzhen 518107, China

**Keywords:** image generation, generating adversarial network, diffusion model

## Abstract

Intelligent defect detection technology combined with deep learning has gained widespread attention in recent years. However, the small number, and diverse and random nature, of defects on industrial surfaces pose a significant challenge to deep learning-based methods. Generating defect images can effectively solve this problem. This paper investigates and summarises traditional defect generation and deep learning-based methods. It analyses the various advantages and disadvantages of these methods and establishes a benchmark through classical adversarial networks and diffusion models. The performance of these methods in generating defect images is analysed through various indices. This paper discusses the existing methods, highlights the shortcomings and challenges in the field of defect image generation, and proposes future research directions. Finally, the paper concludes with a summary.

## 1. Introduction

The manufacturing industry is a crucial element of a country’s comprehensive national strength, and a foundational industry of its economy. The quality of manufactured goods is a determining factor in the country’s competitiveness and reputation. During the manufacturing production process, quality of the produced goods can be affected to different levels due to technical and environmental factors. Surface defects are often the most recognisable sign of product quality deterioration.

**Figure 1:**
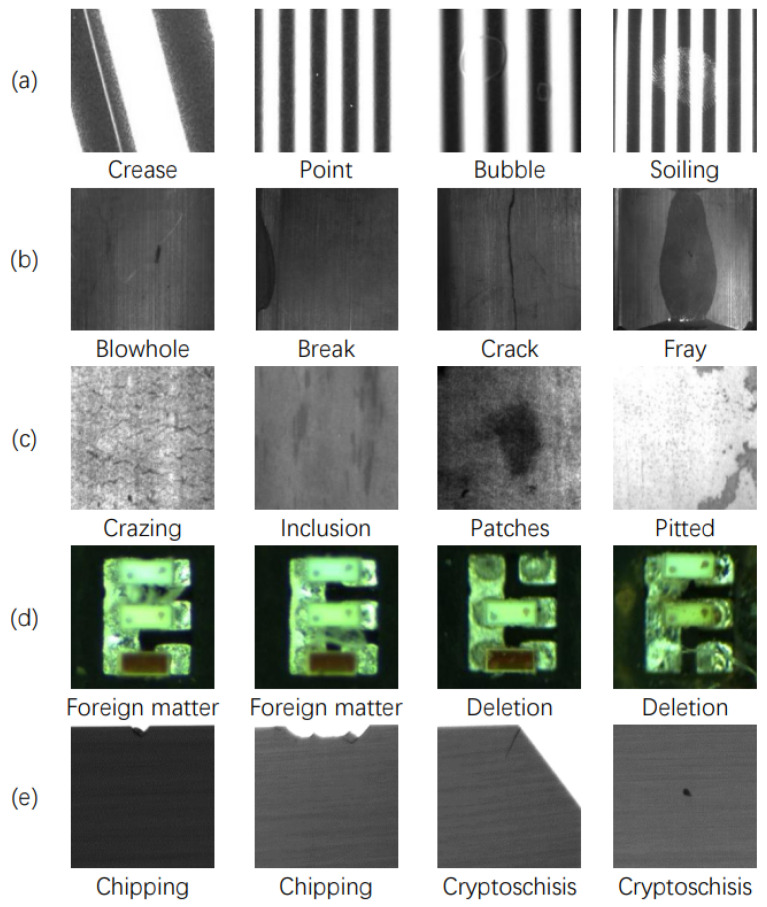
(**a**) Structured light image of polarizer surface defects. (**b**) Examples of https://github.com/abin24/Magnetic-tile-defect-datasets (accessed on 20 August 2023) magnetic tile surface defects. (**c**) Images of surface defects on steel [2]. (**d**) Images of surface defects on MiniLED. (**e**) Images of surface defects on solar silicon wafers.

Surface defects can refer to missing, damaged, or deformed product characteristics when compared to normal specimens [1]. Industrial surface defects are a general term for a range of surface defects that can appear on industrial product materials and affect product quality. Based on surface stiffness, industrial surface defects can be divided into two categories: surface defects of flexible products and surface defects of rigid products. Characteristically, surface defects of flexible products show bubbles, wrinkles, and point defects among others, whereas surface defects of rigid products usually present cracks, chippings, abrasion, and scratches. Common defects are depicted in Figure 1.

Inspection of industrial surface defects has the potential to enhance product quality while also reducing manufacturing costs and time. Despite this, a large number of industrial products continue to rely on manual inspection, which makes it difficult to effectively control time, cost, and quality. This situation is not conducive to the further development of the manufacturing industry. Therefore, the development of intelligent defect detection technology is essential.

Intelligent defect detection refers to the automated or semi-automated inspection of industrial product surfaces using machine vision technology. This technology can be categorised into traditional method-based and deep learning-based methods. Traditional method-based defect detection techniques typically require manual design of feature extractors and classifiers. However, they are often difficult to adapt to complex and changing industrial scenarios and lack generalisation ability and robustness. Deep learning-based defect detection techniques can use neural networks to automatically learn defect features from a large amount of data. This leads to efficient and accurate defect detection.

However, deep learning-based defect detection techniques also encounter several challenges. Firstly, deep learning models require a large number of labelled, high-quality defect samples for training. However, obtaining these samples can be challenging due to reasons such as cost and difficulty in collecting industrial defect data.

Moreover, industrial surface defects often have a long-tailed distribution, with some defect types accounting for a large proportion of the total number of defects while others account for a small proportion. As a result, defect samples of these types are usually rare, posing a major challenge to the detection task. Deep learning-based defect detection methods face several challenges. To overcome these issues in practice, data augmentation techniques are often employed when the number of defect samples is inadequate to cover all the defect distributions they represent. Traditional data augmentation methods, such as geometric transformations, rotations and flips, are commonly utilised in deep learning models to enhance the model’s generalisation ability. However, this method can solely enhance the existing data, making it complicated to supplement other faulty patterns with additional information. To resolve this, a new approach has been proposed to supplement the defect sample data by constructing synthetic defect images. The technique involves simulating and synthesising defect images with various types, shapes, sizes, and locations utilising artificial intelligence techniques, thereby augmenting the existing dataset. This technique can introduce more unknown defect information into the deep learning model, thereby increasing the accuracy of defect detection. Several studies [3,4,5,6,7] have demonstrated that the generated defect data can significantly augment the accuracy of defect detection. Consequently, the technique enhances the training effect and generalisation ability of deep learning models, and its use has expansive prospects and potential application in the manufacturing industry.

In the field of image generation, traditional methods are commonly used to create images via image processing techniques. Within deep learning, generative models fall into several categories including autoregressive models [8], variational autoencoders [9], stream-based models [10], GAN (generative adversarial networks) [11], and diffusion models [12]. Since the autoregressive model requires pixel-by-pixel image generation, it has either poor performance or high cost for large-scale image generation. While the variational autoencoder is not as adept as GAN at generating detailed images due to its lack of the adversarial game concept, it generates blurred images. In contrast, the streaming model is computationally expensive and complex. However, GAN is exceptional in its performance and realistic image generation through the concept of adversarial generation, and has gained significant attention in the field of image generation since its proposal in 2014. Generative adversarial networks (GANs) have been widely applied in texture synthesis [13,14], image super-resolution [15,16,17,18], image restoration [19,20,21,22], image translation [23,24,25], image editing [26,27], and multimodal image synthesis [28,29,30]. A diffusion model is a type of generative model that produces high-quality images from noise. The denoising diffusion probabilistic model, introduced in 2020 [31], is one such model. Diffusion models are interpretable because they are a type of latent variable model. Unlike other latent variable models such as variational autoencoder (VAE), the inference process of diffusion models is typically fixed. Additionally, the latent space of diffusion models is interpretable, allowing us to understand how changes in the latent variables impact the generated outputs. This can be beneficial in applications where we wish to control distinct features of the generated outputs. The intensive research on diffusion models has made it one of the most significant areas of computer vision in recent times. Diffusion models can generate higher-quality images than GANs, without requiring a discriminator model. However, they are slower and more computationally intensive to train. Although there have been many studies on diffusion models, our investigation indicates that no one has yet used them in the field of generating defect images on industrial surfaces.

**Figure 2:**
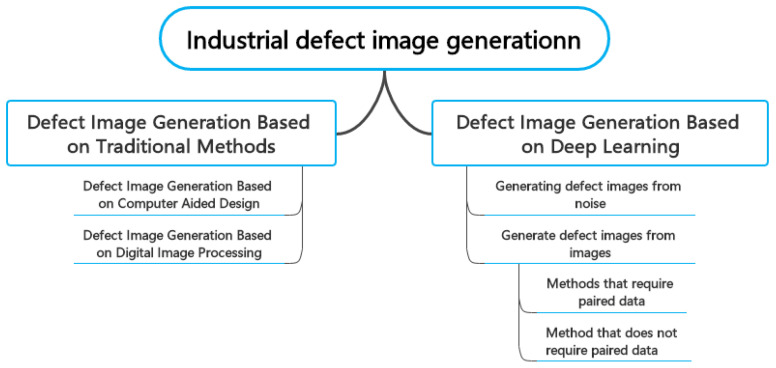
Classification map of defective image generation methods.

**Figure 3:**
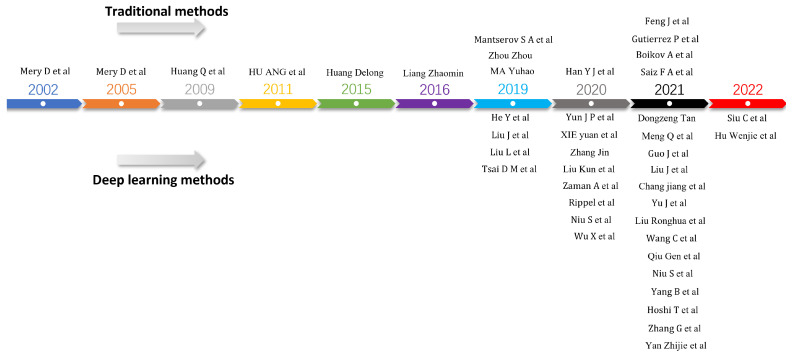
Development process of defective image generation methods [3,4,5,6,7,32,33,34,35,36,37,38,39,40,41,42,43,44,45,46,47,48,49,50,51,52,53,54,55,56,57,58,59,60,61,62,63,64,65,66,67].

Currently, there is limited research available on the topic of industrial surface defect generation. Figure 2 shows the classification of these works. This paper categorises the methods of industrial defect image generation into traditional and deep learning methods. Specifically, traditional methods include generation methods based on computer-aided techniques and digital image processing. Deep learning methods are classified into two categories: generating defect images from noise and generating defect images from images, where generating defect images from images can be further divided into methods that require paired data and methods that do not require paired data. This paper, to our knowledge, is the first review that categorises and summarises both traditional and deep learning methods for defect image generation. As shown in Figure 3, traditional-based defect image generation methods had relevant works earlier than deep learning-based methods which only began to emerge in 2019. Subsequently, the number of papers published on both traditional-based and deep learning-based defect image generation methods shows a consistent upward trend, suggesting an increased interest in this field. Consequently, it would be beneficial to provide a research summary and analysis of the related works in this field, which can serve as a reference for future researchers in the field of intelligent defect inspection.

This paper provides a comprehensive review and analysis of the methods for generating industrial surface defect images, which are essential for improving the performance and robustness of intelligent defect detection systems.The main contributions of this paper are as follows: Firstly, we propose a novel classification scheme for the methods of industrial defect image generation, based on whether they use traditional or deep learning techniques, and whether they require paired data or not. We also provide a detailed introduction and comparison of the existing methods in each category, highlighting their advantages, disadvantages, and applications. Secondly, we conduct extensive experiments on three representative adversarial networks (DCGAN, Pix2pix, and CycleGAN) and diffusion model (DDPM) to generate defect images on various public datasets.We evaluate the quality and diversity of the generated images using various metrics, such as FID, IS, PSNR, SSIM, and LPIPS. We also present qualitative results to demonstrate the visual effects of different methods. To the best of our knowledge, this is the first work that establishes a benchmark for industrial defect image generation using deep learning methods. Thirdly, we identify the current challenges and limitations of the existing methods for industrial defect image generation, such as data scarcity, data imbalance, defect diversity, defect realism, and defect controllability. We also discuss the possible future research directions and opportunities in this field, such as incorporating domain knowledge, exploiting multimodal data, enhancing model interpretability, and developing end-to-end networks.

**Figure 4:**
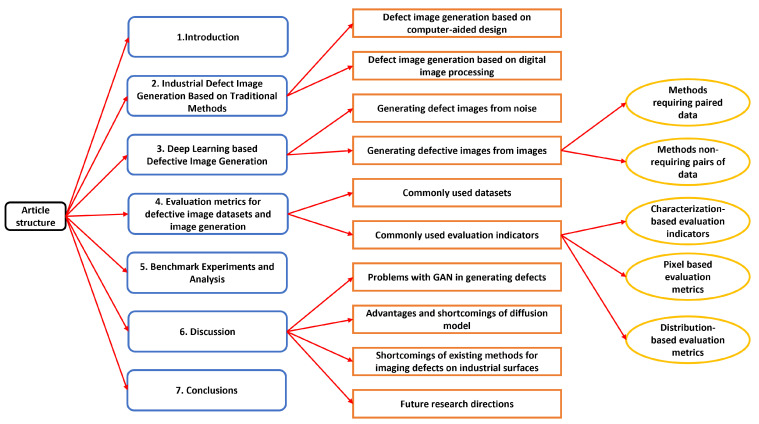
Overall structure of the paper.

The rest of the paper is organised as follows. Section 2 describes the industrial defect image generation based on traditional methods, while Section 3 describes the same based on deep learning. Section 4 discusses commonly used public defect datasets and evaluation metrics for image generation while Section 5 presents comparative experiments on industrial defect image generation using deep learning networks. Section 6 highlights the shortcomings of existing methods and suggests future research directions. Finally, Section 7 concludes the paper. Figure 4 shows the overall structure of the paper.

## 2. Industrial Defect Image Generation Based on Traditional Methods

Traditionally, defect images were generated through conventional methods that enable the control of multiple defect features, such as geometric shapes and positional information, with a certain level of precision. Depending on the modelling requirement, traditional methods can be classified into two types: one is based on computer-aided design, and the other is rooted in digital image processing.

### 2.1. Defect Image Generation Based on Computer-Aided Design

**Figure 5:**
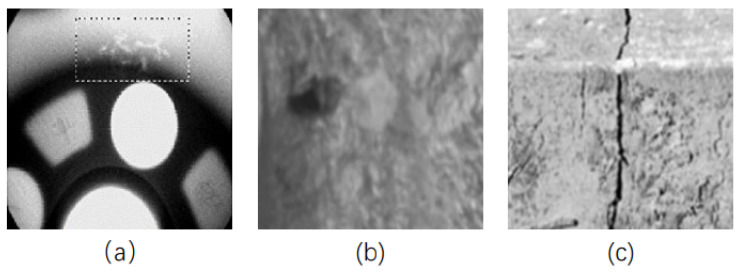
(**a**) Wheel defects. (**b**) Metal parts defects. (**c**) Steel workpiece defects.

Industrial defect images are typically generated by computer-aided design (CAD)-based methods. This can be achieved by constructing CAD models or using software such as 3ds Max, which involves inputting relevant defect geometry and descriptive information. CAD models use techniques such as ray tracing and X-ray imaging to simulate complex 3D objects [68]. These methods are usually applied to rigid materials such as castings, steel, etc., where 3D models are easy to obtain or construct. Figure 5 shows examples of defect images in these typical materials.

**Figure 6:**
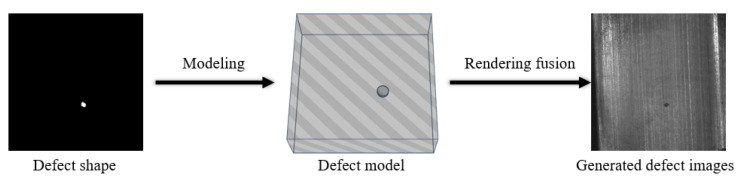
General flowchart for generating defect images based on computer-aided design.

Mery D et al. [32] generated a defect image by projecting a basic 3D model of the defect. They then improved the process by constructing a computer-aided design (CAD) model of the defect, using it as a foreground image and superimposing it on a real X-ray image to generate a synthetic defect [33]. This method models the defects directly and overlooks environmental factors such as brightness and angle during imaging. To capture the genuine environment in defect modelling, it is important to include imaging angle, brightness, and scene. For instance, Saiz F A et al. [34] simulated the potential geometric and photometric changes on the CAD model of the pipe defects to produce a more comprehensive defect image. Boikov A et al. [35] simulated a camera shot of steel through the Blender 3D graphics editor and controlled the texture changes manually by entering parameters. Conversely, Gutierrez P et al. [36] constructed a generalised pipeline for rendering industrial images. They mixed various basic shapes like circles with different types of noise to create diverse defects. In the rendering process, they also factored in lighting and optical systems’ impact on defect formation to more accurately depict the real situation. Virtual reality construction techniques can also be used in defect modelling. For instance, Ma Yuhao [37] employed virtual reality technology to create a virtual experimental environment, develop a sophisticated bevel model, establish a light source library based on self-illuminated materials, use the moving least squares deformation algorithm to deform the 3D model, and, ultimately, batch render the defect images in the virtual environment via 3ds Max software. Figure 6 depicts the overall flowchart of defect image generation using the computer-aided design method.

Generating industrial defect images using CAD requires experts to design, manually, the spatial shape of the corresponding defects. This design process is, however, inevitably limited. Additionally, methods such as CAD modelling and three-dimensional modelling, which require ray tracing and X-ray attenuation calculations, require more computational resources and find it difficult to generate images with irregular defects. Table 1 summarizes computer-aided design-based methods.

### 2.2. Defect Image Generation Based on Digital Image Processing

**Figure 7:**
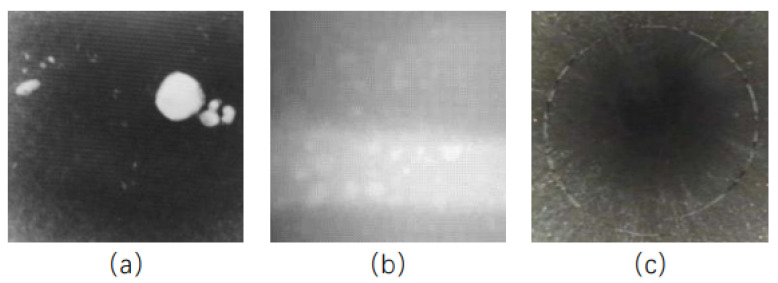
(**a**) Porosity defects in radiographic images of castings. (**b**) Spot defects in X-ray images of castings. (**c**) Pipeline defects.

Methods based on digital image processing typically require specific algorithms to modify the original image to generate images with targeted defects. This approach can overcome the limitations of using CAD based methods such as extended modelling time, greater consumption of computational resources, and fewer types of defects, etc. A significant number of defective images can be created at a lower computational cost, and the resulting images usually exhibit defects such as hole defects, speckle defects or defects with specific geometric shapes, as illustrated in Figure 7.

Defective images can be created by filtering or adding noise to the normal image [38,39]. However, defective images created by simply adding noise lack realism. Hence, image fusion techniques can be employed to superimpose the generated noise on the normal background information, resulting in a more realistic defect image. For instance, Huang Delong [38] generated a sparse defect pattern using Perlin noise, then rendered the defect image in greyscale before finally fusing the rendered defect image with the actual detection background. Liang Zhaomin [40] employed the improved Diamond-Square algorithm to generate elevation data and transformed it into a simulated defective image. Liang then utilised the improved Otsu thresholding method to enhance the diversification and arbitrariness of the defective part and fused the simulated defective image with the background image. Similarly, Zhou Zhou [41] created a spongy defective skeleton, which was later filtered with Gaussian noise and added with Berlin noise. Finally, the wheel core X-ray image was superimposed through image fusion.

**Figure 8:**
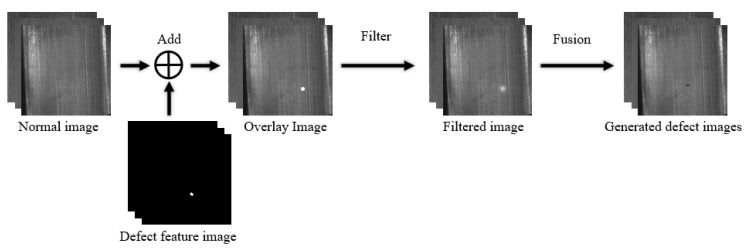
General flowchart for generating defect images based on digital image processing.

Due to the low controllability, it is challenging for humans to achieve the desired defect morphology. Therefore, researchers have incorporated the expert’s priori knowledge to create a system model capable of generating defect features by adjusting parameters. For instance, Huang Q et al. [42] used the defect superposition method of nested stencils with 2D image technology, based on casting defect analysis, to simulate the normal range of casting defects. This involved selecting stencils such as circular, elliptical, or random from a wide range of template libraries and adjusting the intensity and size variation of the stencils through the user’s control parameters. In contrast, Mantserov S A et al. [43] developed a parametric display system for defects by linking the characteristics of defects-such as area, texture, shape, boundary, and HSV-to a set of parameters. By inputting different parameters, the images of various defects can be obtained. Similarly, Han Y J et al. [44] generated binary images with specific defect characteristics using expert knowledge. These images are further merged through the alpha or other methods with defect-free images to create synthetic defect images. Figure 8 depicts the general flowchart of defect image generation based on digital image processing methods.

The process of generating a defective image based on a digital image processing method can also be formulated by the following Equation (1):

(1) $$I_{g}=F\left(L\left(I_{n}+I_{ab}\right)\right),$$

where $I_{g}$ refers to the generated defect image, while *F* and *L* stand for the image fusion and filtering processes, respectively. $I_{n}$ denotes the normal image, whereas $I_{ab}$ represents the defect feature map. The human operation can control $I_{ab}$ by adjusting the size and shape of the defects.

Although this method may produce a considerable number of defect images inexpensively, it is challenging to generate synthetic defect images resembling the actual defects closely, relying solely on digital image processing methods, since the real defect images tend to have complex compositions. Table 2 summarizes methods on digital image processing.

## 3. Deep Learning Based Defective Image Generation

In recent years, several deep learning techniques have been employed in industrial defective image generation due to the rapid advancements in deep learning. For instance, Siu C F et al. [45] transformed simulated defective images into real defective images using a style migration network, whereas Yun J P et al. [46] suggested the use of a method based on a conditional convolutional variational autoencoder (CCVAE) to generate defective images. Moreover, the generative adversarial network (GAN)-based defect image generation method has gained considerable traction in research. Therefore, this section primarily aims to summarise and introduce GAN and its related applications to generate defective images.

GAN is an unsupervised deep learning model that revolves around the optimisation process of a two-player game. The generator generates a fake image to deceive the discriminator by learning the feature distribution of the training data, while the discriminator determines whether the input image is a fake image generated by the generator or a real one. Consequently, the optimisation process of GAN can be represented as a large-scale minimisation problem whose objective function is manifested by Equation (2):

(2) $$\min_{G}\max_{D}V\left(D,G\right)\;=\;E_{x∼P_{\mathrm{data}\;}\left(x\right)}\left[\log\left(D\left(x\right)\right)\right]\;+\;E_{z∼P_{z}\left(z\right)}\left[\log\left(1\;-\;D\left(G\left(z\right)\right)\right)\right].$$

Equation (2) defines the following variables: *G* is the generator, *D* is the discriminator, *z* is the noise input, G(z) is the generated data, D(x) is the probability distribution representing the likelihood that *x* is real data, $P_{data}\left(x\right)$ denotes the distribution of the real data, and $P_{z}\left(z\right)$ denotes the distribution of the generated data. The symbol $P_{z}\left(z\right)$ denotes the distribution of the generated data, while *E* represents the expected value. Equation (2) can be divided into two parts that reflect the generator and discriminator’s respective objective functions, as described in Equations (3) and (4).

(3) $$\max_{D}V\left(D,G\right)=E_{x∼P_{\mathrm{data}\;}\left(x\right)}\left[\log\left(D\left(x\right)\right)\right]+E_{z∼P_{z}\left(z\right)}\left[\log\left(1-D\left(G\left(z\right)\right)\right)\right],$$

(4) $$\min_{G}V\left(D,G\right)=E_{z∼P_{z}\left(z\right)}\left[\log\left(1-D\left(G\left(z\right)\right)\right)\right].$$

Equation (3) seeks to maximise the output of D(x) while minimising the output of D(G(z)). The discriminator’s ability to distinguish between real and generated data is critical. Similarly, in Equation (4), the aim is to maximise the output of D(G(z)) to enable the generator to produce data that deceives the discriminator. This means that the generator should produce data that misleads the discriminator. GAN training can be unstable due to its adversarial nature. In order to create a stable GAN that caters to a variety of tasks, several GAN variants exist. Below is an introduction to some of the more commonly used ones.

- DCGAN [70]: DCGAN provides a convolution-based GAN structure, and DCGAN verifies that discriminators can be used for feature extraction and generators can be used for semantic vector computation.
- Pix2pix [24]: Pix2pix is a conditional GAN that introduces labelled images and performs style migration over paired data.
- CycleGAN [71]: CycleGAN contains two GAN structures, the first GAN is responsible for converting the source domain to the target domain and the second GAN is responsible for converting the target domain to the source domain as a way of transforming specific features of an image.
- SAGAN [72]: SAGAN considers global information at each level and does not introduce an excessive number of parameters, finding a good balance between increasing the feeling field and reducing the number of parameters.
- StyleGAN [29]: StyleGAN uses low to high resolution image generation and can control the visual features expressed at each level by modifying the inputs to that level.

The GAN loss has other common loss functions in addition to the adversarial loss mentioned in Equation (2), and some common losses are described below.

- Reconstruction losses:The reconstruction loss is generally used to train the autoencoder to transform at the pixel level with the loss function shown in Equation (5):
  (5) $$L_{rec}=E_{x,y∼P_{\mathrm{data}\;}\left(x,y\right)}\left[{∥y-De\left(En\left(x\right)\right)∥}_{n}\right].$$
  In Equation (5) ${∥\cdot ∥}_{n}$ denotes the *n*-paradigm, *n* generally takes the value of 1 or 2, *x* is the input data, *y* is the target data, En is the encoder, and De is the decoder.
- Cyclic Consistency Loss: The purpose of cyclic consistency loss is to ensure that the translated samples retain the content of their input samples, which usually requires a pair of GAN structures to compute the loss, whose loss function is shown in Equation (6):
  (6) $$L_{cyc}=E_{x∼P_{data}\left(x\right),y∼P_{\mathrm{data}\;}\left(y\right)}\left[{∥x-G_{2}\left(G_{1}\left(x\right)\right)∥}_{n}+{∥y-G_{1}\left(G_{2}\left(y\right)\right)∥}_{n}\right].$$
  The same ${∥\cdot ∥}_{n}$ in Equation (6) denotes the *n* paradigm, *n* generally takes the value 1 or 2, $G_{1}$, $G_{2}$ denote the two generators, and *x* and *y* denote the source domain data and the target domain data, respectively.
- WGAN losses [73]: The training of GAN is prone to instability and the problem of pattern collapse, the WGAN loss makes the training of GAN more stable by minimising the Wasserstein distance and satisfying the Lipschitz continuity [74], there are many variants of the WGAN loss, of which Equation (7) is the most common WGAN loss:
  (7) $$\min_{G}\max_{D}V\left(D,G\right)=E_{z∼P_{z}\left(z\right)}\left[D\left(G\left(z\right)\right)\right]-E_{x∼P_{\mathrm{data}}\left(x\right)\left[D\left(x\right)\right]}+\lambda_{gp}E_{\hat{x}∼P\left(\hat{x}\right)}\left[{\left({\left|\nabla_{\hat{x}}D\left(\hat{x}\right)|\right|}_{2}-1\right)}^{2}\right],$$
  where $\hat{x}$ refers to uniform sampling along a straight line between a pair of real and generated samples, $\lambda_{gp}$ is a hyperparameter, *x* represents the source domain data, and *z* denotes noise.

There are two types of GAN-based defect generation methods, depending on the type of input data provided to the model. One uses input potential noise vectors, while the other uses input images. Correspondingly, there are two methods—one for GANs requiring paired data and the other for GANs not requiring paired data.

### 3.1. Generating Defect Images from Noise

**Figure 9:**
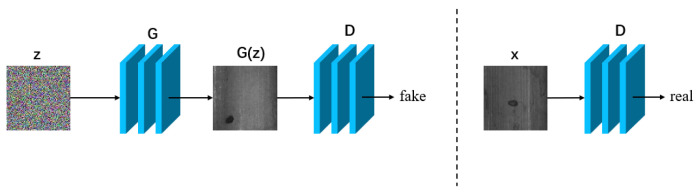
General flowchart for generating defective images based on digital image processing.

To enhance the diversity of the original data and to compensate for a small number of defect samples not covered by the distribution of defects they represent, noise can be added to the input data fed into the network to generate a random image. Figure 9 shows the general flowchart for image generation, where *z* represents noise, *G* denotes the generator, *D* denotes the discriminator, *x* is the real defect image, and G(z) refers to the generated defect image.

**Figure 10:**
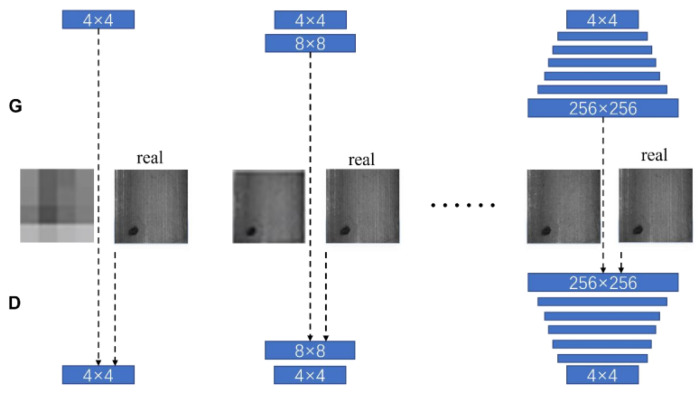
Progressive growth’s training framework.

Xie Yuan [47] employed the DCGAN model structure and included the discriminator’s output dimension to devise a semi-supervised model for generating defective images. At times, converting the noise into different forms can enhance the model’s learning capability. For instance, Jin Zhang [48], convoluted the input noise latent space of the network into a Gaussian mixture model to enhance the image generation network’s learning capacity for limited training samples with inter- and intra-class diversity. However, none of these approaches yield high-resolution images, which are attainable through gradual progressive generation when high-quality images are imperative. For example, Dongzeng Tan [49] proposed the training technique of progressive growth, auxiliary classifiers, and the concept of mutual information maximisation based on DCGAN to accomplish the generation of class-controllable and morphology-adjustable tire defect images, with a specified background condition. Here, progressive growth refers to the model training procedure that commences with a low-resolution image, gradually increasing in resolution, and culminating in the generation of a high-resolution image. The framework illustration is presented in Figure 10.

To create controllable defect images or improve image quality, one can consider adding conditional information and increasing the number of discriminators or classifiers in the GAN. For instance, He Y et al. [50] used cDCGAN to generate defect images, whereas Kun Liu et al. [51] introduced a network structure of NSGGAN to generate defect images with three types of discriminator inputs: the generator-generated image, the real image with defects, and the genuine image without any defects. Meng Q et al. [52] put forward a two-channel generative adversarial network architecture known as Dual-GAN, where its discriminator verifies the authenticity of the entire image and recognises the local faulty areas, tagging the defect types as well. On the other hand, Guo J et al. [53] incorporated two fresh connections on SAGAN to balance the output of the two generators and reflect the disparities in output images between different generators. Meanwhile, Liu J et al. [54] employed conditional GAN and employed feature information obtained from normal samples after passing through the VGG network as the conditional signal to generate defects.

To detect subtle defects or those that have some connection with the background image, an attention mechanism can be introduced to obtain a global receptive field. For instance, Wang C et al. [4] used TransGAN to generate defect images. Hu et al. [55] improved the quality of model generation by introducing a structural similarity loss function and an attention module based on ConSinGAN [75]. When dealing with data imbalance problems, Li W et al. [56] proposed a new generative adversarial network, EID-GANs, to overcome the challenge of extremely imbalanced data enhancement.

Combining label condition information in GAN with reconstructed label information can enhance the quality of generated data. ACGAN [76] incorporates both these approaches. Moreover, Liu J et al. [5] introduced focal loss to ACGAN, which effectively addresses the issue of data imbalance. On the other hand, Chang Jiang et al. [57] enhanced the structure of ACGAN by branching the last layer of the model’s discriminator’s convolutional feature map. This, after spreading and fully connecting it to the output layers of judging truth and category, improved the model’s fitting ability, and made its parameter set smaller. In addition, they also removed the discriminator’s fully connected layer and added the implicit layer. On the other hand, Yu J et al. [6] incorporated an auxiliary feature extractor before the generator of the ACGAN model. This extractor takes the extracted multi-granular features and noise as inputs to the generator.

Images can be generated for different defect data based on their characteristics. For instance, Liu Ronghua [58] captured specific images of industrial defects and encoded their location information. Next, they input the encoded images into StyleGAN to generate new samples, and finally fused the newly generated samples with the location information using an image fusion method. Table 3 summarizes the methods for generating defect images from noise.

### 3.2. Generating Defective Images from Images

Although the potential vector input method may introduce some randomness, the quality of the generated images is often inadequate, with intricate parts being especially difficult to produce. Thus, employing an image as input data can retain some essential information from the original image and produce a defect image that is more lifelike and contains more detailed texture information.

#### 3.2.1. Methods Requiring Paired Data

**Figure 11:**
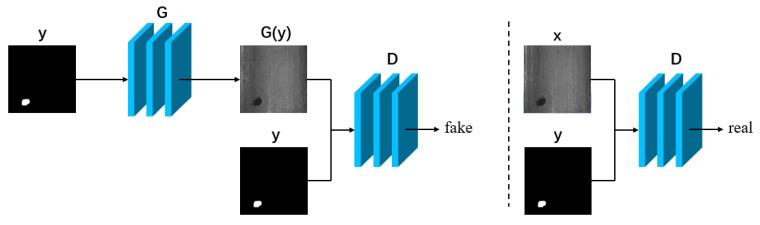
General flowchart for methods requiring paired data.

For paired data, a defect image and a corresponding label map must be available. This ensures that the texture information of the background of the input image is preserved to the highest possible extent, leading to the generation of a high-quality defect image. The overall flowchart for the method is depicted in Figure 11, where *y* denotes the labelled image, *x* denotes the actual defect image, and G(y) denotes the generated defect image. Each pair comprises the images *y* and *x*, and the generator and discriminator are denoted by *G* and *D*, respectively. Pairs of *x* and *y* are used as inputs for the approach.

Zaman [59] generated anomalous bone surface images using a Pix2pix model that shares a similar structure to the previously mentioned figure. Qiu [60] incorporated labeled defect images as inputs to the CycleGAN network to guide the production of defect-free images. Liu [61] proposed a novel framework for generating defect-free samples using GAN, which employed loss functions and a coding and decoding structure to simulate the network and generated localised defects while maintaining the defect-free region unchanged. They also introduced wavelet fusion to the process. On the other hand, Niu [77] designed a GAN-based generative network that uses defect masks to control the location of defect generation based on the characteristics of industrial defects. They further constructed a defect direction vector module that controls the intensity of the generated defects and a defect attention module that enhanced attention to the defect region. Table 4 summarizes the methods that require paired data.

#### 3.2.2. Methods Non-Requiring Pairs of Data

**Figure 12:**
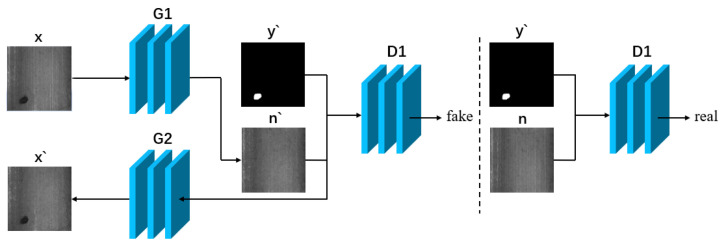
Cyclic direction I: abnormal-normal-abnormal.

**Figure 13:**
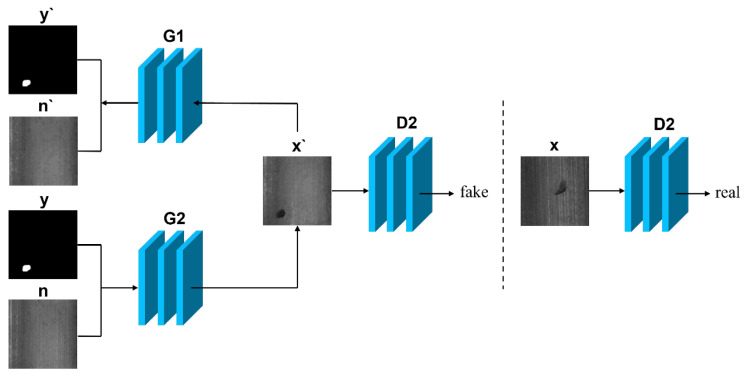
Cyclic direction II: normal-abnormal-normal.

Although methods that utilise paired data have the potential to generate high-quality defect images, in practice, acquiring the necessary paired training data can be challenging and manual labelling is often laborious and time-consuming. In this scenario, an approach similar to CycleGAN that does not require paired data can be employed to produce defect images. Figure 12 and Figure 13 demonstrate the general flow of the method that employs unpaired data.

Generally, methods that do not employ paired data use two loops that can be trained to generate defect images without inputting paired data. Here, *x* denotes the real defect image, *n* represents the real normal image, *y* indicates the labeled image, *x*’ stands for the generated defect image, *n*’ represents the generated normal image, and *y*’ is the generated labeled image. G1 and G2 refer to the two generators, while D1 and D2 correspond to the two discriminators.

**Figure 14:**
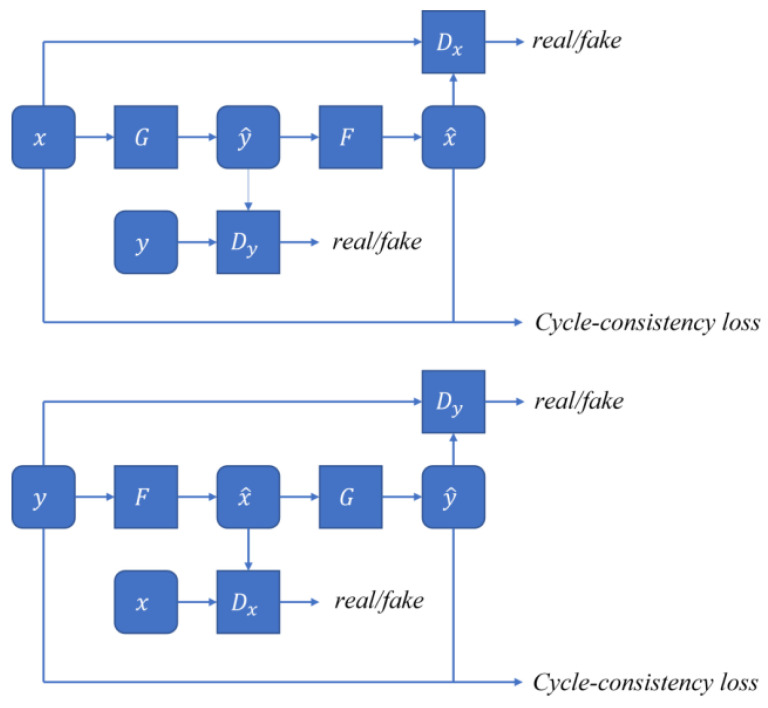
Schematic of loss of cyclic consistency.

AS shown in Figure 14 there are two parts in the cyclic consistency loss: The first part involves transforming an input *x* into $\hat{y}$ using the generator G, then sending both $\hat{y}$ and the real image *y* to the discriminator $D_{y}$. After this, $\hat{y}$ is transformed into $\hat{x}$ using the generator F. Both *x* and $\hat{x}$ are then sent to the discriminator $D_{x}$ again, which completes the first part of the loop. In the second part, we repeat the process with input *y*, which is similar to the first part of the loop. In this context, *y* represents the target image and *x* represents the original image. The cyclic consistency preserves the background information of *x* and only transforms the corresponding transformation domain. The transformation domain refers to the area where *x* and *y* share certain features. For example, zebra and wild horse images share a transformation domain which is the horse. During network training, it is preferred to update the weights in areas where this can be easily implemented. For instance, This ensures that the background information of the images is preserved by updating the weights in the areas where the images share certain common features.

TsaiD M et al. [62] then generated the desired defect images directly by CycleGAN. Rippel et al. [63] improved CycleGAN to a segmented mapping based structure to generate defect images and introduced least squares error and spectral normalisation to improve the stability of training. Niu S et al. [64] added D2 adversarial loss, i.e., adding an additional discriminator loss, within the GAN with a cyclic consistency structure of the GAN within the D2 adversarial loss, i.e., adding the loss of one more discriminator, which allows the model to generate more defect samples. The defect features generated by these methods are more random, and information such as defect masks can also be introduced into the network to generate the specified defect shapes. For example, Yang B et al. [7] proposed a conditional generation GAN, which controls the shape, size, angle and other features of defects through defect masks, and also introduces the structure of cyclic consistency, while Wu X et al. [3] proposed a ResMask GAN network for generating defect images, which uses random masks as part of the network inputs to control the locations and sizes of defect regions, and also designed two discriminative networks. They also designed two discriminators, one for judging the authenticity of the defective part of the image and the other for judging the authenticity of the whole image.

**Figure 15:**
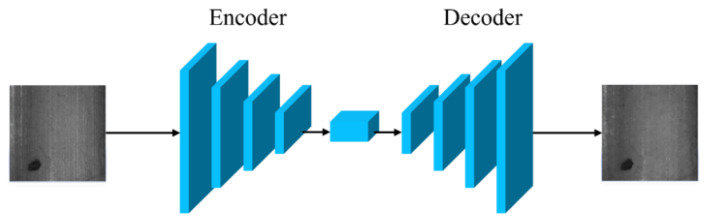
Self-encoder structure diagram.

**Figure 16:**
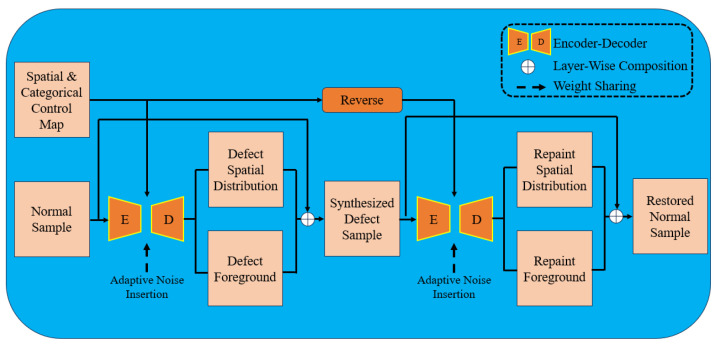
Structure of Defect-GAN proposed by Zhang G et al.,zhang2021defect.

A self-encoder is a semi-supervised or unsupervised neural network which contains two parts, an encoder and a decoder; after the encoder learns the characterisation information of the input data, the corresponding characterisation information can be restored to the input information by the decoder, and its structure is shown in Figure 15:

This structure can sufficiently extract the feature information of the image, which is very suitable for the image generation field, so the introduction of the self-encoder structure in the GAN framework can help in the generation of faulty images. For example, Hoshi T et al. [65] added encoder and decoder structures to their generator and discriminator structures within the framework of CycleGAN network and added an attention mechanism between them, while Zhang G et al. [66] added a weight-sharing self-encoder structure to the CycleGAN model, added adaptive noise to increase the diversity of generated defect samples in training, and added adaptive noise to increase the diversity of generated defect samples in training. By adding adaptive noise to increase the diversity of error samples generated, and adding a spatial and categorical control map to control the category and location of errors in the model, Figure 16 shows the model structure of the method.

It is also possible to generate only for defective regions, as Yan et al. [67] did in the Starganv2 [75] model, which incorporates the Unet network to keep the background morphology unchanged when generating the defective image, and also incorporates the cyclic consistency loss and the target mask loss as a way to constrain the generation of defective samples. Table 5 summarizes the methods that do not require paired data.

## 4. Evaluation Metrics for Defective Image Datasets and Image Generation

### 4.1. Commonly Used Datasets

Unlike datasets such as ImageNet [78] and CelebA [79] for classical vision tasks, there is no unified, large-scale public dataset for industrial surface defect datasets, and the number of samples, positive and negative ratios, and complexity vary widely between datasets.The following Table 6 shows common industrial surface defect datasets.

### 4.2. Commonly Used Evaluation Indicators

The evaluation metrics of faulty image generation are basically the same as those of GAN, focusing on two main aspects. One is to evaluate the quality of the generated results, i.e., the similarity, and the other is to evaluate the diversity of the generated results. Since the traditional loss function is difficult to evaluate the generated defective images such as human eyes, some quantitative indices are used to complement the evaluation.

#### 4.2.1. Characterisation-Based Evaluation Indicators

- *IS*

The *IS* (Inception Score) metric [86] is one of the commonly used evaluation metrics for GANs, and the main idea is to evaluate the quality of the generated images using an image category classifier, where the classifier is Inception Net-V3, which has been pre-trained on the ImageNet dataset.

The GAN-generated image x is fed into the Inception Net-V3 network to obtain a 1000-dimensional vector y. For this vector y, one of the 1000 dimensions should be close to 1 and the others should be close to 0, which reflects the quality of the image generation, and for all generated images, if it is uniformly distributed in all categories, it reflects the good diversity of the generated images. *IS* of the calculation formula is as follows:

(8) $$IS=\exp(\frac{1}{N}\sum_{i=1}^{N}D_{KL}(p\left(y|x^{i}\right)\left|\right|\hat{p}\left(y\right))),$$

where *N* is the number of images generated, $D_{K}L$ is the KL scatter, $p(y|x^{i})$ is the probability distribution of belonging to all classes for a given image *x*, and $\hat{p}\left(y\right)$ is the edge probability, which is the average of the vector of p(y|x) over all generated images *x*.

However, because Inception Net-V3 is an Image Net-based classifier, its output error is larger for other data that does not belong to the Image Net category, in addition, *IS* cannot detect the problem of model collapse, i.e., if the categories of the generated images are evenly distributed in 1000 dimensions, but the images generated in each category are the same, at this time IS still gives a higher score, and similarly *IS* cannot detect the phenomenon of overfitting the model, which are the drawbacks of the *IS* metrics.

- *FID*

*FID* (Fréchet Inception Distance) [87] is also used with the help of the Inception Net-V3 network, but the 2048-dimensional vectors before its fully connected layer are taken as the features of the image, and the Fréchet Distance of the Gaussian distribution of the real and generated images in the feature space is calculated, and the smaller the value, the better the quality of the generated images, and the formula for calculating *FID* is as follows:

(9) $$FID=\left|\right|u_{r}-u_{g}{\left|\right|}^{2}+tr\left(\Sigma_{r}+\Sigma_{g}-2{\left(\Sigma_{r}\Sigma_{g}\right)}^{1/2}\right),$$

where $u_{r}$ is the feature mean of the real image, $u_{g}$ is the feature mean of the generated image, tr is the trace, $\sum_{r}$ is the covariance matrix of the real image, and $\sum_{g}$ is the covariance matrix of the generated image.

The advantage of *FID* is that it solves the error problem that arises when the input data from *IS* to the generative model is very different from ImageNet. In addition, *FID* is robust to noise and has low computational complexity. The disadvantage is that since the distance is computed according to a Gaussian distribution, the extracted image features do not necessarily correspond to the Gaussian distance.

- *LPIPS*

LPIPS (learned perceptual image patch similarity) [88] is a criterion for evaluating image similarity, the evaluation metric is calculated by extracting features through a neural network and then calculating their feature distance, unlike *FID*, the basenet of LPIPS does not necessarily have to be Inception Net-V3, it can be selected from within other typical neural networks for extracting features, and the formula of LPIPS is shown below:

(10) $$d\left(x,x_{0}\right)=\sum_{l}\frac{1}{H_{l}W_{l}}\sum_{h,w}\left|\right|w_{l}\cdot \left({\hat{y}}_{h,w}^{l}-{\hat{y}}_{0h,w}^{l}\right){\left|\right|}_{2}^{2},$$

where *x* and $x_{0}$ are the two input images, *l* denotes the *l*th layer of the neural network, ${\hat{y}}_{h,w}^{l}$ and ${\hat{y}}_{0h,w}^{l}$ denote the feature information of the two input images after passing through the lth layer of the neural network and normalised, respectively, and $w_{l}$ stands for a weighting layer, and · is the dot product.

The advantage of LPIPS is that it is more in line with human perception, but the disadvantage is that it can only reflect the similarity of the images, not the diversity of the images.

- *KID*

*KID* (Kernel Inception Distance) [89] also calculates the representation distance using the Inception Net-V3 network but, unlike *FID*, *KID* calculates the square of the maximum mean difference between the Inception representations, i.e., the square of the *MMD* to measure the difference between the two sample sets, which is calculated as follows:

(11) $$MMD_{u}^{2}\left(X,Y\right)=\frac{1}{m(m-1)}\sum_{i\neq j}^{m}k\left(x_{i},x_{j}\right)+\frac{1}{n(n-1)}\sum_{i\neq j}^{n}k\left(y_{i},y_{j}\right)-\frac{2}{mn}\sum_{i=1}^{m}\sum_{j=1}^{n}k\left(x_{i},y_{j}\right),$$

where *m* is the sample size of the generated image and *n* is the sample size of the real image.

The advantage of KID is that there is an unbiased estimate of the cubic kernel; compared to the FID metrics it is a better match to human perception. The disadvantage of KID is that it is more computationally intensive.

#### 4.2.2. Pixel-Based Evaluation Metrics

- *PSNR*

*PSNR* (Peak Signal-to-Noise Ratio), which is used to measure the difference between two images, is calculated as follows:

(12) $$PSNR=10\times \log_{10}\left(\frac{{\left(2^{n}-1\right)}^{2}}{MSE}\right),$$

where *n* is the number of bits in the image and MSE is the mean square error of the two images.

The advantage of *PSNR* is that it is easy to calculate and understand and can give a rough indication of the quality of the image; the disadvantage is that the quality of the image reflected in the *PSNR* is sometimes not entirely consistent with the results observed by the human eye.

- SSIM

SSIM (structural similarity) [90] measures the similarity of an image in terms of its three aspects: brightness, contrast and structure, where brightness is calculated as follows:

(13) $$l\left(x,y\right)=\frac{2u_{x}u_{y}+C_{1}}{u_{x}^{2}+u_{y}^{2}+C_{1}},$$

(14) $$c\left(x,y\right)=\frac{2\sigma_{x}\sigma_{y}+C_{2}}{\sigma_{x}^{2}+\sigma_{y}^{2}+C_{2}},$$

(15) $$s\left(x,y\right)=\frac{\sigma_{xy}+c_{3}}{\sigma_{x}\sigma_{y}+C_{3}},$$

where $u_{x}$, $u_{y}$, $\sigma_{x}$ and $\sigma_{y}$ are the local mean, variance and covariance of *x* and *y* respectively, and $C_{1}$, $C_{2}$ and $C_{3}$ are constants that avoid setting the divisor to 0. The SSIM is symmetric, and the SSIM reaches its maximum value of 1 when the two images are identical.

The advantage of SSIM is that it improves the shortcomings of PSNR and is more in line with human visual characteristics, but its disadvantage is that there is no way to evaluate the image well in the face of non-structural distortions such as rotations, translations, and deflations in the image.

- *Sharpness Difference*

*Sharpness difference* and *PSNR* metrics are calculated in a similar way, but they are more concerned with the difference in sharpness information between images, which is calculated using the following formula:

(16) $$Sharpness\;Difference_{I,K}=10\log_{10}\left(\frac{Max_{I}^{2}}{\frac{1}{N}\sum_{i}\sum_{j}\left|A-B\right|}\right),$$

(17) $$A=|I_{i,j}-I_{i-1j}\left|+\right|I_{i,j}-I_{i,j-1}|,$$

(18) $$B=|K_{i,j}-K_{i-1}j|+|K_{i,j}-K_{i,j-1}|,$$

where *I*, *K* are the two images respectively, MAX is the maximum possible pixel value of the image, the larger the sharpness difference value, the smaller the difference in sharpness between the two images, the better the quality of the generated image.

The advantage of sharpness difference is that it can reflect whether the generated image has similar sharpness to the original image, while its disadvantage is that it focuses only on sharpness and lacks other information considerations, making it difficult to fully reflect the quality of the generated image.

#### 4.2.3. Distribution-Based Evaluation Metrics

- *MMD*

*MMD* (Maximum Mean Discrepancy) [91] is the Maximum Mean Difference, a Hilbert space measure of the difference between two distributions, and its basic defining formula is as follows:

(19) $$MMD\left[F,p,q\right]:=\overset{sup}{f\in F}\left(E_{p}\left[f\left(x\right)\right]-E_{q}\left[f\left(y\right)\right]\right).$$

This equation is a mapping function that maps variables to a higher dimensional space and then finds the expected difference between the variables of the two distributions after the mapping.

The advantage of *MMD* is that it does not have to rely on additional parameters to judge the distance between two distributions, while its disadvantage is that it is not able to detect the difference in distribution between natural and adversarial data [92].

- 1-NN

**Figure 17:**
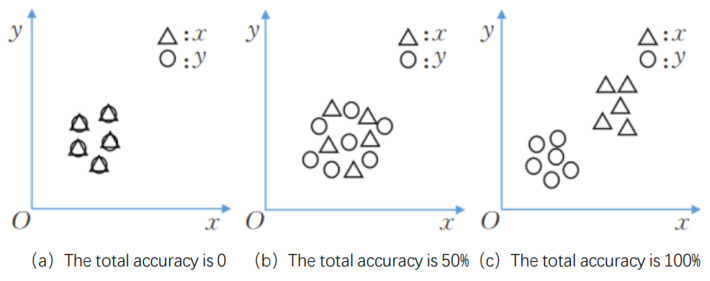
Comparison of total correct rates [93].

1-NN (1-Nearest Neighbour Classifier): by comparing the thinking, calculate the probability distribution of the training dataset and the generated dataset and compare; if the smaller the difference between the two results indicates that the GAN generation performance is better, Figure 17 shows the change in the correct rate of each test data on the 1-NN. The larger the difference between the distribution of the correct rate is the higher the performance of the GAN generation, that is, worse.

The advantage of 1-NN is that it is easy to understand and implement and does not require parameter estimation, but its disadvantage is that it is insensitive to the case of model collapse in the generative model.

## 5. Benchmark Experiments and Analysis

Traditional methods are usually based on modelling or require expert a priori knowledge, making it difficult to apply them directly to public datasets. In addition, most of the deep learning methods investigated in this paper do not open source their code or describe the details of their implementation process, so in order to provide reference examples of defect generation methods, this paper will go from the three GAN-based generation methods and the SD(Stable Diffusion) pre-training model based on the Latent Diffusion Model (LDM), respectively, to the SD pre-training model based on the Latent Diffusion Model (LDM), The experiments are analysed from three GAN-based methods and the SD model based on the latent diffusion model (LDM), namely the method with inputs as potential vectors (StyleGAN), the method with inputs as paired data (Pix2pix), the method with inputs as unpaired data (CycleGAN), and the method with a SD model based on LoRA fine-tuning. The dataset required for this experiment should contain normal and abnormal samples as well as the labelled images corresponding to the abnormal samples, considering the size and authenticity of the dataset, this paper adopts the Magnetic-tiledefect datasets (MT Defect Dataset) as the dataset for this experiment.

Diffusion modelling is a recent research hotspot within the field of image generation, and it can be said that diffusion generation models have raised the bar in the field of image generation to a new level, especially when referring to models such as Imagen [94] and the Latent Diffusion Model (LDM) [95]. All of these models use the powerful text comprehension capabilities of contrastive language-image pre-training (CLIP) [96], which is based on contrastive text-image pairs, and the high-fidelity image generation capabilities of diffusion models to achieve the mapping from text to image. Therefore, we believe that the great potential of diffusion models in the field of image generation can be applied to the generation of defect images of industrial surfaces, which can generate high quality defect images while controlling the features of defect image generation, so in this paper we have included two diffusion models to generate defect images in the experiments.

The stabilised diffusion model introduces potential space into the generation process by transforming images into potential vectors to reduce computational complexity and increase efficiency. In this way, it can significantly reduce the time and economic cost of training while maintaining the quality of generation. Although the stabilised diffusion model has a strong image generation capability, its training dataset is mainly focused on natural scenes and cannot be directly applied to industrial surface defect image generation tasks, and the resource cost of re-training is usually prohibitively high. The parameter-efficient fine-tuning (PEFT) method can solve this problem very well, and PEFT can efficiently adapt the model to different downstream application tasks without fine-tuning all parameters of the pre-trained model. There are three main categories of existing PEFT methods: adapter tuning [97,98], prefix tuning [99], and LoRA (low-rank adaptation) [100]. Adapter tuning introduces additional computation by adding a layer of adapters on top of the original model, which leads to the inference delay problem; while prefix tuning adds a layer of adapters on top of the original model, which leads to the inference delay problem. Prefix tuning is also difficult to optimise and its performance varies non-monotonically with the size of the trainable parameters. The fine-tuning method freezes the original model parameters, represents them, and constrains their updates by low-rank decomposition, which can greatly reduce the memory and storage resource consumption during fine-tuning without introducing additional inference latency. To impose controllable conditional constraints on the diffusion model, ControlNet [101] proposes a method to enhance SD by adding conditional inputs such as graffiti, edge mapping, segmentation mapping, pose keypoints, etc. to the text-to-image generation process. It can make the generated image closer to the input image, which is a great improvement over the traditional image-to-image generation method, in the industrial surface defect generation task, the mask label can be used as a control condition for the image sampling process to guide the direction of the diffusion model generation, by controlling the region and morphology of the mask, so as to obtain the diversity generation dataset with mask, without manual labelling.

**Figure 18:**
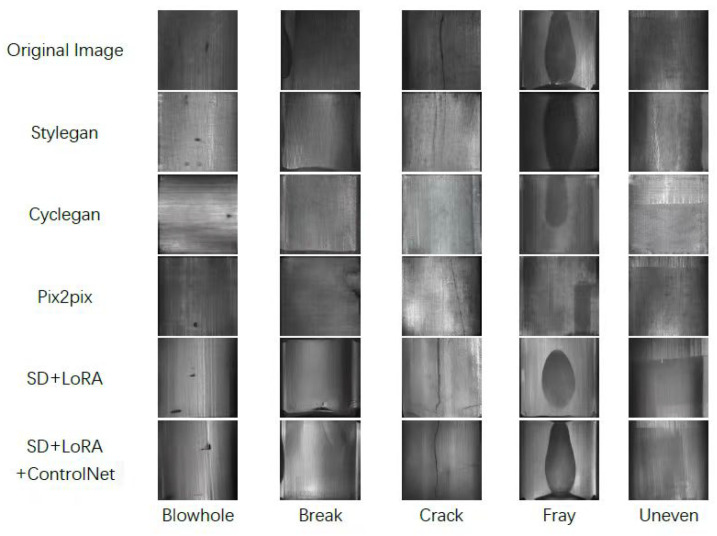
Generated defect images.

Five defect images are generated by five methods on the dataset that has been doubled by geometric transformations, and the results are shown in Figure 18:

Observing the defect images generated by the five methods in Figure 18, it can be seen that the defect images generated by SD+LoRA have the best background texture and defect details and the highest degree of image reproduction, and the conditional constraints can be added to the sampling and diffusion process through the mask-labelled image pairs after the addition of ControlNet to make the generative model able to control the defect generation area and shape, thus obtaining surface defect images with pixel-level labelling. The defect images generated by Pix2pix also have good background texture and defect details, but for some defects with small sample size, the generation effect is average and the generated images are not diversified enough, and the defect morphology and location are relatively homogeneous, while Cyclegan is not as good as Pix2pix in terms of background texture information. Stylegan’s background texture information is the worst, and has some distance from the background of the real image.

**Figure 19:**
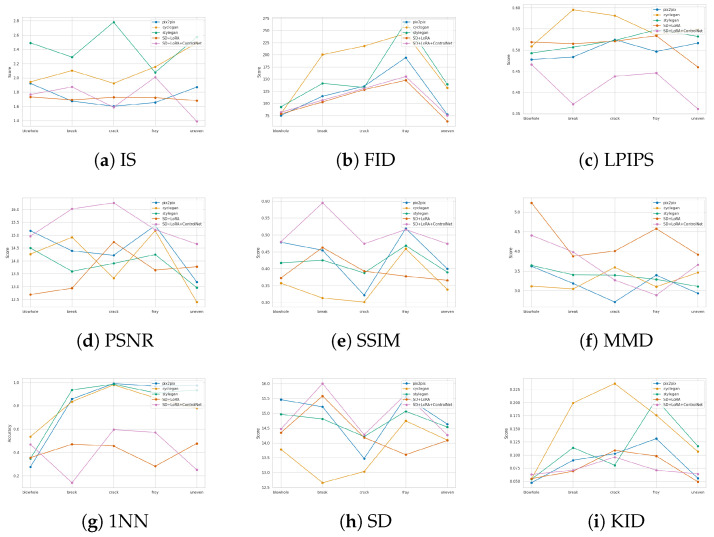
Chart of results for commonly used evaluation indicators.

Common evaluation metrics are used to calculate the defect images generated by these methods separately, as shown in Figure 19:

As can be seen from Figure 19, the diffusion model based SD + LoRA generation method basically performs the best, and is superior to the GAN network in the vast majority of evaluation metrics, and this phenomenon is most obvious in the Fray defects with the smallest number of samples, which is mainly attributed to the powerful generalisation ability of the large model, And only a small number of samples are needed to fine-tune the model, and then we can achieve a good generation effect, in the calculation of image feature similarity FID, LPIPS, KID, IS indicators, SD + LoRA method and SD + LoRA + ControlNet method is basically the same, these two methods to generate the image is basically in the same sample space. While in the calculation of PSNR, SSIM and Sharpness Difference metrics of the construction and pixel differences between images, the method of SD+LoRA+ControlNet is significantly better than the method of SD+LoRA, which is mainly due to the fact that ControlNet is able to achieve controllable generation of defective regions and shapes in measuring the metrics of a single generated image than the random generation of SD+LoRA. In the GAN network, the Pix2pix method, which uses paired data for training, basically performs the best.This is because when paired data are used for training, the model can obtain information such as the texture, features and location of the defects of the image, so as to generate a higher quality defect image. For the two methods, Cyclegan and Stylegan, Cyclegan will perform better than Stylegan in calculating the feature similarity index of the image, For both Cyclegan and Stylegan, when the defects are relatively small or have a relatively regular shape such as bubbles and grey, Cyclegan will perform better than Stylegan in calculating the feature similarity metrics of the image, this is because when the data are not trained, the labelled images are still artificially synthesised and there is some gap between them and the actual defect shapes. Stylegan, on the other hand, learns the original data distribution directly to generate defect images, and therefore generates large or irregular defects that are more similar to the original image defects than Cyclegan. For the distribution-related 1-NN and MMD metrics, the defect samples generated by the SD+LoRA method can be well maintained in the original sample space without the phenomenon of overfitting, and the 1-NN classification accuracy hovers around 40–50%, whereas the training of the GAN will suffer from the phenomenon of overfitting, and the distribution of the generated samples will be more like the distribution of the original data, so the classification accuracy will not be around 50% and, at the same time, the value of the MMD will also be smaller.

In order to make these methods more intuitive, we also carried out the actual classification task, by the classification accuracy to determine the quality of the generated defect images, the MT Defect Dataset normal images and five kinds of defect images according to the 6:2:2 divided into the training set, the validation set and the test set to perform a six-classification task, and then add the same number of the three methods generated by the same number of the five kinds of defective images to the training set, in the Resnet101 model under the co-training of the 100 epoch, select the validation set of which the classification of the highest accuracy of the model to go to the test set to test the accuracy of the accuracy of the test set, as shown in Table 7.

Classification accuracy after defect image augmentation using all five methods improved compared to the original data, with the method using SD + LoRA + ControlNet having the highest classification accuracy, and pix2pix ahead of a number of GAN networks such as Stylegan. Stylegan had better accuracy than Cyclegan on the validation set and worse accuracy than Cyclegan on the test set, and the classification results are generally consistent with the metrics analysis. This suggests that the good quality of the generated defect images, such as detail information, background texture information and sample distribution, is an area that can influence their classification accuracy for subsequent classification tasks. Generating high quality defect image samples can bring some performance improvement for the subsequent classification and detection tasks, and the improvement is more obvious in the scenarios with smaller sample size.

## 6. Discussion

### 6.1. Problems with GAN in Generating Defects

Although GAN has been widely used in the field of image generation, there are some problems with both the GAN itself and its application to the task of generating defects on industrial surfaces:

- Mode collapse

The GAN may go into an unstable state, causing the generator to output only a certain type of image and the discriminator to be unable to distinguish between true and false, in which case the GAN may produce duplicate or similar images that do not cover the true distribution of the data and produce images that lack diversity.

- Multimodal distribution learning difficulty

The GAN may enter an unstable state that causes the generator to output only one type of image and the discriminator to be unable to distinguish between true and false, in which case the GAN may produce duplicate or similar images that do not cover the true distribution of the data and produce images that lack diversity.

- Long training time

GANs require a large amount of data and computational resources to train, and the training process may oscillate or fail to converge, often requiring multiple adjustments to the model hyperparameters to make the model converge, resulting in long training times.

- Difficulties in training

The objective function of GAN is a min-max problem, which needs to balance the adversarial relationship between the generator and the discriminator, and there is no clear evaluation index to measure the generation effect, which makes the GAN not easy to train, and at the same time, the training of the GAN often requires larger data samples, and industrial surface defects tend to have a long-tailed distribution, with more samples of normal samples and certain defects, and the number of defects in some categories is extremely small, even only a few in the extreme environment, which further makes it difficult to train the GAN.

- Difficulty in generating high resolution images

In industrial environments, high-resolution images are often required because they can contain more information and details, but GANs may lose some important features or produce some artefacts and noise during the generation process [31].

### 6.2. Advantages and Shortcomings of Diffusion Model in Generating Defects

The diffusion model is a probabilistic-based deep generative model that converts a data distribution into a simple prior distribution by gradually adding noise to the data, and then samples and recovers the data distribution from the prior distribution by learning an inverse denoising process. Diffusion models have demonstrated superior performance in image generation tasks, outperforming traditional methods such as adversarial generative networks (GAN) and variational autoencoders (VAE) [12]. The diffusion model also beats GAN networks in evaluation metrics in the area of image synthesis [102].

The stable diffusion model is a pre-trained model trained on 512 × 512 images of the LAION-5B dataset, which is advantageous for generating high-resolution images. This is due to the fact that the potential diffusion model maps the image to potential space using VQ-VAE2 and uses sampling methods such as DDIM [103] to speed up the sampling process and reduce the number of sampling steps, thus achieving a reduction in computational cost without sacrificing image quality.From the quality of the images generated in the experiments, it can be seen that the diffusion model can be applied to industrial surface defect image generation, which has better stability and interpretability compared to the traditional GAN.

However, for high-resolution image generation tasks, time and economic cost are the main factors limiting the application of the diffusion model. At present, the generation speed of the diffusion model is still not superior to that of the GAN. In terms of model structure, the sampling speed of the diffusion model is relatively slower than that of GAN, and the sampling of GAN only requires one neural network feedforward. The diffusion model requires T-step feedforward. In the experiments, the average time of Pix2pix, Cyclegan, Stylegan model to generate an image of 256 × 256 size is 0.02925 s, 0.07725 s, 0.04969 s, respectively, while the stable diffusion model + LoRA takes 2.28 s to generate an image and the stable diffusion model + LoRA + ControlNet takes 3.67 s to generate an image. Moreover, the StyleGAN-T model proposed by Sauer et al. [104] is about 30 times faster than the stable diffusion model in the inference stage.

### 6.3. Shortcomings of Existing Methods for Imaging Defects on Industrial Surfaces

Despite many advances, there are still some shortcomings of the current methods for defect imaging on industrial surfaces:

- Small number of datasets and defective images

Existing datasets of industrial surface defects are relatively small, and there is a lack of a common large canonical industrial defect dataset as a benchmark for defect model generation. In addition, all existing deep learning methods require real defect images for training, but in practice the number of certain types of defects is very small, single sample or even zero sample, but the existing methods pay less attention to this point.

- Weak generalisation of methods

Most of the current defect image generation methods are specific to the characteristics of a particular defect image, and once you change a defect image, you have to design a new network or structure, etc., so there is a need to study methods with more generality.

- Incomplete evaluation indicators

Existing commonly used evaluation metrics generally calculate the gap of a certain aspect to judge the quality of the generated image, but this is not fully applicable to defect images. Defect images are different from general image generation tasks, with problems such as complex defect morphology, large defect size range, and interference from background information, so there is a lack of an index that can evaluate the quality of generated defect images in a more objective and comprehensive way.

### 6.4. Future Research Directions

By combining the current research progress in defect image generation, it is possible to look forward to future research directions.

- Based on traditional methods

Traditional methods have the advantages of speed and interpretability over deep learning methods, so it is also interesting to investigate how to generate images of multiple defect types with similarity to real defects using traditional methods.

- Deep learning-based approach

Diffusion models have better stability and interpretability than generative adversarial networks (GANs), which are usually based on two neural networks playing off each other, one to generate samples and the other to discriminate between real and generated samples. This approach typically requires a large amount of training data and computational resources, and suffers from training instability and pattern collapse. The diffusion model, on the other hand, is based on traditional mathematical models and has better stability and interpretability. It does not require a large amount of training data and can learn and predict from a small amount of data. In addition, the diffusion model can control its sensitivity and robustness by adjusting the model parameters to adapt to different data distributions and noise situations.

In addition, in practice, the number of a certain defect image may be single digit or even only one, this time how to go through one or more images to generate defect images need to be studied, there is related work with a single defect image generation [55], the future can be combined with the relevant structures and methods in small-sample learning or zero-sample learning to perform the defect image generation.

## 7. Conclusions

With the advent of the intelligent manufacturing era and the rapid development of machine vision and deep learning, data have become a valuable resource, and how to supplement datasets by synthesising defects has gradually become one of the new research problems under the assumption of limited real defect data.

The aim of this paper is to provide researchers with more research ideas on defect image generation, so it summarises the past and present defect image generation methods and classifies them into two categories: traditional methods and deep learning methods. It focuses on introducing and analysing the GAN-based defect image generation methods in deep learning, tests three different inputs based on GAN on a public dataset, applies the diffusion model to the defect image generation of industrial surfaces for the first time, discusses the characteristics of these methods in conjunction with the experimental results, and, finally, in the Section 6, it discusses the shortcomings of the existing methods as well as points out possible research directions for the future.

In general, the performance of the model in the downstream task can be improved by image generation and, compared with the traditional image generation methods, the deep learning-based image generation methods will be more generalised and stochastic. There are still some problems that have not been solved by the current deep learning-based image generation methods, which need to be researched further.

## Figures and Tables

**Table 1:** Summary table of computer-aided design-based methods.

Literature	Published	Brief Description	Advantage	Disadvantage
[32]	2002	Synthesise a defect image by projecting a simple 3D model defect	Simulation of spherical bubble defects in castings, with simple 3D modeling	Single type of defects that can be simulated
[33]	2005	Construction of defective foreground image by CAD, superimposed on normal image	Complex geometric defects of any size and orientation can be simulated anywhere in the casting	Requires expert a priori knowledge and is computationally resource intensive and time consuming
[34]	2021	Defect modelling via CAD, with geometric and photometric transformations also incorporated	Can better simulate the real environment to model	Few types of defects detected, only 2D surface modelling
[35]	2021	Simulate shooting scenes with the Blender 3D graphics editor	Multiple defect types can be generated	High consumption of computing resources and long time
[36]	2021	Constructed a generalised pipeline for dyeing and rendering industrial images	Defect image simulation by physical sensors or based on statistical measurements is possible	Each individual use case requires a model of the available parameters
[37]	2019	Construction of randomised defects by virtual reality techniques and least squares methods	Randomly generate various types of simulated bevel defects	Simple structure and single function of 3D model

**Table 2:** Summary table of methods based on digital image processing.

Literature	Published	Brief Description	Advantage	Disadvantage
[38]	2015	Generate sparse defect masters using Perlin noise and render and blend them with normal images in grayscale	Introducing semantic-driven techniques to build a controlled sparse defect generation program that accepts natural language inputs	Fusion algorithms only target places with uniform grey values, and semantic-driven algorithms may lead to inaccurate localization
[40]	2016	Generation of elevation data by improved Diamond-Square algorithm and conversion to simulated defect images	Generate simulated shrinkage defects of arbitrary size and shape	A single type of defect is targeted, the operation is slow, and the location of defect generation is not randomised enough
[39]	2021	Defect colour and shape generation via Gaussian kernel distribution and random graphs	Generates defect images with complex backgrounds	The process of generating defect images is cumbersome
[41]	2019	Gaussian filtering on sponge defective skeleton and fusion with normal image	Without relying on existing defect samples, sponge like shrinkage defects with random size, length, and shape can be generated	The method is only targeted at specific defects and cannot be generalized to other defects
[42]	2009	Defect simulation by defect overlay method with nested stencils in 2D image technology	The user can create many different defects and add them to the actual casting image	X-ray images only
[43]	2019	Construction of a defect parameter display system that associates defect characteristics with a set of parameters	User-adjustable parameterised defect shape	Parameters for defects need to be adjusted repeatedly
[44]	2020	Fusion of binary images with expert prior knowledge with normal images via alpha, etc	Defects can be generated by adding a corresponding defect binary map	Requires a great deal of expert a priori knowledge
[69]	2011	Defect image generation by Gaussian noise	The geometry of the defects in the simulation process varies a lot, and the greyscale approximates the real defects	Cannot generate defective images in colour

**Table 3:** Summary table of methods for generating defect images from noise.

Literature	Published	Brief Description	Advantage	Disadvantage
[4]	2021	Defect Image Generation by TransGAN	Introducing an attention mechanism to improve the quality of synthetic samples	Training samples need to be manually selected and annotated, which is time-consuming
[5]	2021	Adding focal loss to the ACGAN network	Good solution to the problem of unbalanced data	Limited categories of defects generated
[6]	2021	An auxiliary feature extractor was added in front of the generator of the ACGAN network	Considering optimisation terms in the loss function and integrating the discriminator with lightweight convolution reduces the computational cost	The training time is still quite long
[47]	2020	Adding the output dimension of the discriminator in DCGAN	Simplifies the degree of parameter redundancy as well as the difficulty of model optimisation	Only low resolution defect images can be generated
[48]	2020	Complicating the latent space of network input noise into a Gaussian mixture model	Improving the learning ability of generative networks for a limited number of training samples with polygyny	The quality of the defective images generated only performs well in a particular metric or approach
[49]	2021	Training DCGAN using Progressive Grow with auxiliary classifiers and ideas based on mutual information maximisation	Mitigates training instability and improves the generation quality of defective images through stepwise generation	Insufficient diversity of the generated defect images, which does not take into account the distribution characteristics of the defect images
[50]	2019	Defect Image Generation by Conditional GAN	The discriminator can be used as a binary classifier in addition to determining whether an image is generated or not	Low resolution of generated images
[51]	2020	The NSGGAN model is proposed with three inputs to its discriminator	Improved model representation of positive samples and diversity of generated samples through negative sample bootstrapping strategy	Need to have a certain number of negative samples to train
[53]	2021	Two additional connectors have been added to the SAGAN	Mutual learning between the two generators is possible through the addition of connectors	The resolution of the generated defect images is low and the scale of the hyperparameters needs to be adjusted
[54]	2019	Extracting features to act as conditional information for conditional GAN via VGG network	Specific defect types can be generated from condition information	Conditional information is first extracted by the VGG network and is divided into two stages to generate the defective image, which is time-consuming
[55]	2022	Structural similarity loss function and attention module introduced on the basis of ConSinGAN networks	By combining structural similarity loss and self-attention module, the problem of industrial defect image distortion and texture training difficulty is solved	Easy distortion of defective edges in the generated image
[56]	2022	The generator is guided to learn the characteristics of the outliers by subtracting them from the cropping region of the generated instances	Addressing extreme data imbalances, while also proposing a new assessment methodology	Requires manual cropping of outlier datasets, which does not take into account the defect contrast information on the generated images
[57]	2021	Improved network structure of AC-GAN	Solve the category misalignment problem of the original AC-GAN and generate more realistic images with steel defects	Validated only for low-resolution images
[58]	2021	Specific defect images are intercepted based on the location information, encoded and fed into the StyleGAN network	Editability of the generated image is achieved by hidden layer coding transformations	Difficulty in obtaining rich simulated defects with insufficient samples

**Table 4:** Summary table of methods requiring paired data.

Literature	Published	Brief Description	Advantage	Disadvantage
[59]	2020	Image generation via Pix2pix network	No need to use complex architectures or expensive training processes	Generation effects are strongly influenced by the quality and quantity of data
[60]	2021	Corresponding defect labeled images were added to the CycleGAN network	Synthetic defects similar to real defects were synthesised with little additional supervision	The labels produced are not sufficiently randomised
[61]	2019	Proposing a new defective sample generation framework and region training strategy	Introducing an image local translation training strategy to solve the blurring problem of synthesised images caused by the loss of high-frequency information	Loss of high-frequency information in non-deficient areas still exists
[77]	2021	A GAN network designed according to the characteristics of industrial defects	Control of the intensity and shape of the defective region through defect masks and defect direction vectors	The relationship between defect intensity and image features needs to be further analysed

**Table 5:** Summary table of methods that do not require paired data.

Literature	Published	Brief Description	Advantage	Disadvantage
[3]	2020	Proposed ResMask-GAN network based on cyclic consistency	You can control the shape of the defects generated by the masks	Requires manual creation of labeled images
[7]	2021	Proposing a GAN network using defect masks as conditional information	Controlled synthesis of defects with different shapes, severities, scales, etc	Depth of information on defects not considered
[62]	2019	Defect Image Generation by CycleGAN	Generation of synthetic defect images from unpaired data	The quality of the generated images leaves a lot to be desired
[63]	2020	Improvement of segmented mapping-based structures on CycleGAN with introduction of least squares error and spectral normalisation	You can specify where defects are generated without using pairs of data	The colour consistency of the generated defect images could be improved
[64]	2020	Incorporating D2 against loss inside a GAN with a cyclic consistency structure	High-quality defective samples can be synthesised by making full use of non-defective samples	The additional loss introduced is more training time consuming
[65]	2021	Incorporating self-encoder structures with attention mechanisms on CycleGAN	Added attention module to obtain global information	Poor resolution of generated defect images
[66]	2021	Self-encoder structure with weight sharing added to CycleGAN	Provides flexible control over the location and type of defects generated	More complex model structure
[67]	2021	Introduced Unet network with cyclic consistency loss and target mask loss	Introducing non-defective samples as auxiliary training reduces the amount of model dependence on defective samples	Lack of a very rigorous interpretation of image styles, with some subjectivity in the criteria used to classify them

**Table 6:** Common industrial surface defect datasets.

Name	Number of Images (Sheets)	Whether There Are Positive and Negative Examples	Availability of Labeled Images
Magnetic tile-defect datasets [80]	1344	Yes	Yes
NEU surface defect database [2]	1800	No	Yes
Kolektor Surface Defect Dataset [81]	399	Yes	Yes
RSDDs dataset [82]	195	No	Yes
MVTec Anomaly Detection [83]	5354	Yes	Yes
DAGM2007 [84]	3450	Yes	Yes
AITEX [85]	245	Yes	Yes

**Table 7:** Classification accuracy.

Method	Val_Accuracy	Test_Accuracy
Resnet101	0.7173	0.7556
Resnet101 + Stylegan	0.8143	0.8143
Resnet101 + Cyclegan	0.7669	0.8236
Resnet101 + Pix2pix	0.8159	0.8540
Resnet101 + SD + LoRA	0.8292	0.8661
Resnet101 + SD + LoRA + ControlNet	0.8518	0.8829

## Data Availability

Data sharing is not applicable to this article as no new data were created or analysed in this study.
